# Large Built‐in Fields and Tunable Ferroelectricity in Composition‐Graded ScAlN Thin Films Deposited by Reactive Sputtering

**DOI:** 10.1002/advs.202500611

**Published:** 2025-04-25

**Authors:** Tai Nguyen, Anirban Ghosh, Thang Duy Dao, Maja Koblar, Goran Drazic, Nikolai Andrianov, Iurii Nesterenko, Sanjay Nayak, Joaquin Miranda, Andreja Bencan Golob, Mohssen Moridi, Marco Deluca

**Affiliations:** ^1^ Microsystem Division Silicon Austria Labs GmbH Villach 9524 Austria; ^2^ Electronic Ceramics Department Jožef Stefan Institute Ljubljana 1000 Slovenia; ^3^ Department of Materials Chemistry National Institute of Chemistry Ljubljana 1000 Slovenia

**Keywords:** wurtzite ferroelectrics, Sc_x_Al_1‐x_N, ferroelectric shape memory, composition‐graded thin films, built‐in field

## Abstract

The control of the coercive field and growth of abnormally oriented grains (AOGs) in wurtzite ferroelectric Sc_x_Al_1‐x_N thin films is crucial for piezoelectric and ferroelectric applications. However, elevated Sc concentrations generally result in significant AOG growth and high leakage current, degrading piezoelectric and ferroelectric properties. Here, compositionally graded Sc_x_Al_1‐x_N layered structures grown by sputtering are explored to effectively limit the AOG growth, compared to the 8–10% typically observed in conventional films, without requiring process optimization. Notably, a large built‐in electric field of ≈0.45 MV cm^−1^, nearly 2.5 times larger than in graded PbZr_x_Ti_1‐x_O_3_ (PZT) and BaSr_x_Ti_1‐x_O_3_ (BST) systems, is achieved. This built‐in field induces unique features, including horizontal shifts in polarization‐electric field hysteresis loops, bi‐stable states in capacitance‐voltage characteristics, and highly asymmetric electrostrain behavior. Unlike other graded systems, this large built‐in field in the graded Sc_x_Al_1‐x_N films arises primarily from polarization and chemical gradients. These findings offer a simplified, scalable and CMOS‐compatible strategy to overcome the AOG challenges and tune ferroelectric properties of Sc_x_Al_1‐x_N thin films, providing the path for potential applications in advanced photonic and MEMS devices with improved performance and low‐power consumption.

## Graphical abstract

This study explores composition‐graded Sc_x_Al_1‐x_N thin films grown by large‐scale and CMOS‐compatible sputtering that can effectively suppress abnormally‐oriented grains. Importantly, their ferroelectric properties can also be tuned owing to large built‐in fields induced by chemical/polarization gradient. These built‐in fields induce asymmetric behaviors in polarization‐voltage hysteresis loops, capacitance‐voltage, and electrostrain curves.

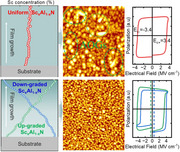

## 1 Introduction

Exotic physical properties often arise from anisotropic interactions between ordering parameters. These phenomena can be achieved through interface engineering, enabling the tailoring of ferromagnetic and ferroelectric properties in complex oxides and nitrides. For instance, the interfacial interaction resulting from the coupling of magnetic moments at the interface between paramagnetic LaNiO_3_ and ferromagnetic LaMnO_3_ thin films gives rise to magnetic exchange bias.^[^ ^1^ ^]^ Similarly, in ferroelectrics, the anisotropic interaction between defect dipoles and ferroelectric dipoles induces strong ferroelectric domain pinning, leading to highly asymmetric electrostrain characteristics with a giant strain exceeding 1%.^[^ ^2^, ^3^ ^]^ Alternatively, compositionally graded thin films, in which composition gradually varies across the film thickness, have shown significant promise not only for engineering strain to achieve defect‐free structures and bandgap control in Al_x_Ga_1‐x_N^[^ ^4^ ^]^ and In_x_Ga_1‐x_As,^[^ ^5^ ^]^ but also for introducing novel phenomena in ferroelectrics.^[^ ^6^, ^7^, ^8^, ^9^ ^]^ In compositionally graded PbZr_x_Ti_1‐x_O_3_ (PZT) and BaSr_x_Ti_1‐x_O_3_ (BST) thin films, a strong built‐in electric field is generated due to a spatial polarization gradient, along with a strain‐gradient‐induced flexoelectric field, imitating the anisotropic interactions between electric dipoles.^[^ ^6^, ^7^, ^8^ ^]^ This built‐in field results in horizontal shifts in the ferroelectric hysteresis loop, large susceptibility, and high dielectric tunability. Hao et al. demonstrated that the polarization switching speed is significantly improved in compositionally graded Hf_1‐x_Zr_x_O_2_ thin films, which is more than twice as fast as those of composition‐uniform thin films.^[^ ^10^ ^]^

AlN and Sc_x_Al_1‐x_N are key materials that have attracted increasing attention for a wide range of applications, including photonics,^[^ ^11^ ^]^ sensors,^[^ ^12^ ^]^ actuators,^[^ ^13^ ^]^ energy harvesters,^[^ ^14^, ^15^ ^]^ and memory devices.^[^ ^16^, ^17^, ^18^ ^]^ Interest in Sc_x_Al_1‐x_N has particularly increased following the discovery of its giant piezoelectric coefficient in 2009^[^ ^19^ ^]^ and ferroelectricity in 2019.^[^ ^20^ ^]^ However, two critical challenges hinder the integration of Sc_x_Al_1‐x_N into practical applications, including the presence of abnormally oriented grains (AOGs) and the high coercive field (2–5 MV cm^−1^). Although increasing Sc content can reduce the coercive field, this also introduces challenges, such as higher leakage current due to narrower bandgaps^[^ ^21^ ^]^ and Sc segregations at the grain boundaries.^[^ ^22^ ^]^ Furthermore, higher Sc concentrations tend to promote the formation of a large number of AOGs, which negatively affect piezoelectric and ferroelectric performance.^[^ ^23^, ^24^, ^25^ ^]^ For instance, Liu et al.^[^ ^24^ ^]^ found that 6% of AOGs in the total volume of the film can degrade the effective coupling coefficient of the resonator from 13.6 to 11%, leading to a 10% decrease in the filter bandwidth. Therefore, optimizing the growth of high Sc‐content Sc_x_Al_1‐x_N films is highly demanding and requires considerable effort. Despite this, to date, the underlying mechanism of the AOG formation has not yet been fully elucidated. For example, Fichtner et al. correlated the AOG growth to internal stress within the film^[^ ^26^ ^]^ or the presence of AOGs at the film‐substrate interface,^[^ ^25^ ^]^ while Sandu and co‐workers linked it to Sc segregation at the grain boundaries.^[^ ^27^ ^]^ This lack of comprehensive understanding of the AOG growth consequently leads to substantial challenges for the fabrication of AOG‐free Sc_x_Al_1‐x_N thin films.

While a recent study by Wang and co‐workers explored the potential of compositionally graded Sc_x_Al_1‐x_N films grown by molecular beam epitaxy (MBE) for multi‐level memory, highlighting a 30% reduction in the working voltage and a 50% improvement in the tuning window,^[^ ^28^ ^]^ our study presents a different focus. Using a scalable, 8‐inch‐capable, and CMOS‐compatible sputtering technique, we offer a straightforward and effortless approach to effectively suppress AOGs and engineer the dielectric and ferroelectric properties of Sc_x_Al_1‐x_N. This method achieves nearly complete AOG inhibition, even at high Sc concentrations, and without requiring additional process optimization. The sputtered graded Sc_x_Al_1‐x_N films exhibit unique characteristics, including a giant built‐in field of 0.45 MVcm^−1^ and highly asymmetric electrostrain behavior, distinguishing them from previously reported graded systems such as Sc_x_Al_1‐x_N by MBE, PZT and BST. By demonstrating both scalability and CMOS compatibility, our graded Sc_x_Al_1‐x_N thin films could provide a versatile ferroelectric platform for potential applications in low‐power and high‐performance photonics, RF, non‐volatile memory, and MEMS devices.

## 2 Results and Discussion

**Figure 1:**
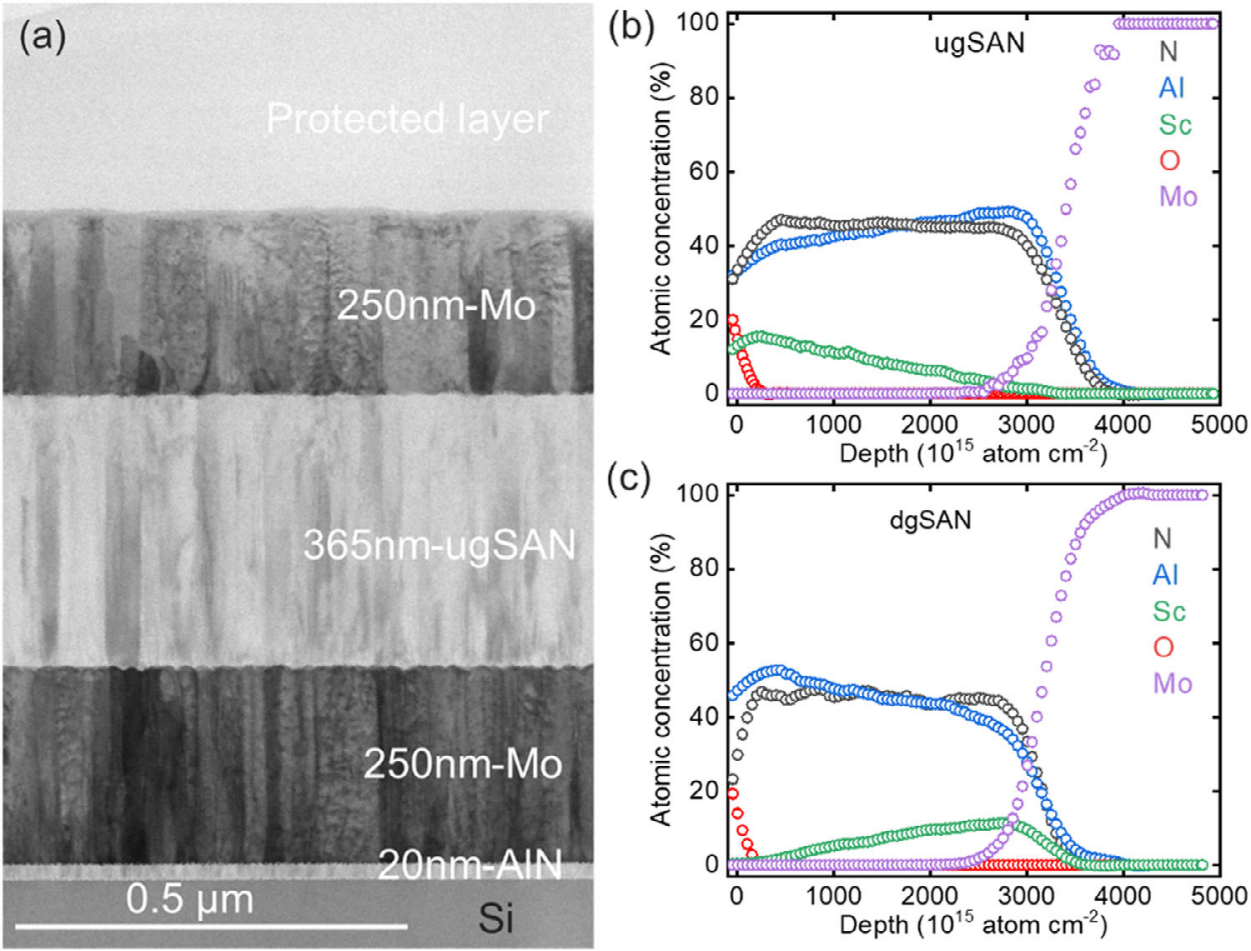
a) Cross‐sectional BF‐STEM image of ugSAN sample showing the different layer stack grown on Si wafer, ToF‐ERDA data of b) ugSAN and c) dgSAN samples.

We prepared five distinct variants of the functionally thin films on 8‐inch Si wafers: a pure AlN, Sc_0.15_Al_0.85_N (15SAN), a film with increasing (compositionally up‐graded) Sc concentration from 0 to 28% (ugSAN), a film with decreasing (compositionally down‐graded) Sc concentration from 28 to 0% (dgSAN) and the highest‐Sc‐concentration Sc_0.28_Al_0.72_N (28SAN) film. **Figure** **1**a shows a cross‐sectional bright‐field scanning transmission electron microscope (BF‐STEM) image of the ugSAN film, which is representative of AlN, 15SAN, dgSAN, and 28SAN samples. Initially, a 20‐nm‐thick AlN seed layer was deposited, followed by a 250‐nm‐thick Mo thin film serving as a bottom electrode. Subsequently, AlN, 15SAN, ugSAN, dgSAN, and 28SAN thin films, each with a thickness of ≈365 nm, were deposited. Finally, a 250‐nm‐thick Mo thin film was deposited as the top electrode layer (cf. Experimental Section).

Time‐of‐Flight elastic recoil detection analysis (ToF‐ERDA) was conducted on identical layer stacks without the Mo top electrode layer to determine the exact composition and gradient profile throughout the films. Figure 1b,c present the ToF‐ERDA data for the ugSAN and dgSAN samples. For simplicity, those of the AlN, 15SAN, and 28SAN samples are shown in Figure S1 (Supporting Information). The Sc concentration was determined to be ≈15 and 28% relative to the overall cation (Sc + Al) concentration for the 15SAN and 28SAN samples, respectively. Therefore, the Sc concentration in the ugSAN (dgSAN) thin film steadily increased (decreased) from 0 (28%) to 28% (0%) across the samples, as clearly shown in Figure 1b,c.

**Figure 2:**
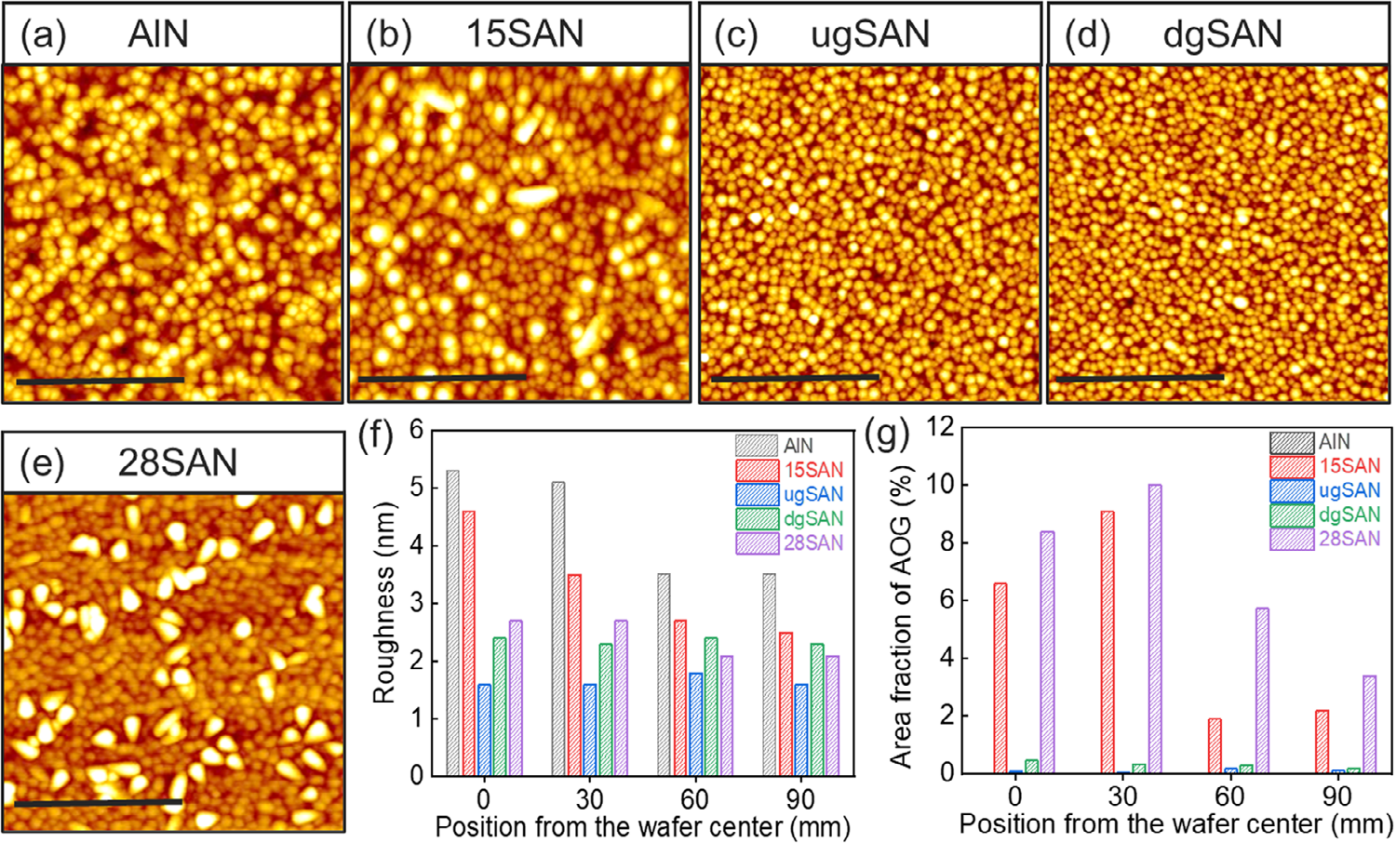
500 nm × 500 nm AFM micrographs of a) AlN, b) 15SAN, c) ugSAN, d) dgSAN, and e) 28SAN samples acquired at the center of 200 mm Si wafer; f) RMS roughness and g) the area fraction of AOGs at different positions of 200 mm wafer. All scale bars are set to 250 nm.

**Figure** **2**a–e presents atomic force microscopy (AFM) micrographs taken at the center position of 200 mm wafers, the root‐mean‐square (RMS) roughness, and the estimated area fraction of AOGs of the five samples. AFM images acquired at the 30, 60, and 90 mm positions radially from the center are shown in Figure S2 (Supporting Information). Figure 2a reveals that the AlN surface consists of pebble‐like grains, resulting in a high surface roughness of ≈5.3 nm. The roughness of AlN improves slightly moving away from the center, with a value of 3.5 nm measured at the 90 mm position, as shown in Figure 2f. A similar trend is seen in the 15SAN and 28SAN samples. Namely, the surface roughness for the 15SAN sample is 4.6 nm at the center and 2.5 nm at the 90 mm position, while those of the 28SAN sample are ≈2.7 nm at the center and 2.1 nm at the 90 mm position. Interestingly, the surface roughness of the ugSAN and dgSAN thin films is significantly lower than that of the uniform thin films, measuring ≈1.6–1.8 and 2.3–2.4 nm, respectively, and remains uniform throughout the entire 200 mm wafers. Notably, while AOGs are clearly visible in the 15SAN and 28SAN thin films, they are strongly suppressed in the ugSAN and dgSAN thin films across the entire wafer. The area fraction of AOGs was carefully analyzed based on their height and shape, as presented in Figure 2g. The detailed analysis can be found in Figure S3–S6 (Supporting Information) for the 15SAN, ugSAN, dgSAN, and 28SAN samples, respectively. A large number of AOGs is observed at the center position for the 15SAN and 28SAN thin films, with the area fractions of ≈8.3 and 10%, respectively. These fractions drop significantly to ≈2.2 and 3.3% at the edges. In contrast, the proportion of AOGs is very minimal throughout the entire 200 mm wafers for the ugSAN and dgSAN thin films, falling below 0.2 and 0.5%, respectively.

The occurrence of AOGs in ScAlN thin films can be attributed to several factors such as the nucleation of different orientation at the film‐substrate interface,^[^ ^23^, ^25^, ^29^ ^]^ processing parameters.^[^ ^30^, ^31^, ^32^ ^]^ Sandu and co‐workers proposed that the AOG growth in ScAlN thin films is induced by the complexion formation, triggered by the segregation of Sc at grain boundaries (GBs).^[^ ^27^ ^]^ Alloying Sc into AlN film induces local structure distortion, and the wurtzite structure can stabilize up to a Sc content of ≈20%, depending on the substrate.^[^ ^33^ ^]^ However, AOGs have already occurred at much lower Sc contents of 10–16%.^[^ ^27^ ^]^ The occurrence of AOGs was later attributed to the segregation of Sc at grain boundaries, where Sc concentration is enriched compared to grains. The segregation of Sc to grain boundaries is driven by the local structural distortion that serves as nucleation sites for the AOG formation. In thin film growth, the Sc segregation is strongly impacted by the lattice mismatch between the film and substrate. As the film grows thicker, strain relaxation leads to the formation of more misfit dislocations. Hirata et al.^[^ ^33^ ^]^ found that the tendency to the phase separation can be suppressed if the film thickness is below the critical thickness. Reducing the lattice mismatch can increase the critical thickness. In 15SAN and 28SAN, the high concentration of Sc and the misfit strain due to the lattice mismatch accelerate the Sc segregation to grain boundaries, thus promoting the formation of AOGs. In contrast, the suppression of AOGs observed in both ugSAN and dgSAN can be explained through several key mechanisms including the misfit strain. The gradual change in Sc content smoothly adjusts the lattice parameters throughout the film layers. This smooth variation reduces the misfit strain due to the lattice mismatch between adjacent layers and minimizes the possible defect sites for Sc segregant accommodation, thus suppressing the formation of AOGs. In the graded films, the thickness of each individual layer was fixed ≈20 nm. According to Hirata et al. model,^[^ ^33^ ^]^ such a thin layer with a nearly perfect lattice mismatch compared to the former layer can effectively prevent the phase separation. In addition, we further performed EDX analysis on two nearby grains along the entire ugSAN film, as shown in Figure S10 (Supporting Information). It can be clearly seen that no Sc segregation to GB throughout the ugSAN film can be detected within the limits of EDX analysis. This observation strongly supports that the growth of AOGs can be greatly reduced due to the suppression of Sc segregation in the graded films. For a more comprehensive understanding, we believe that a study of thermodynamic simulation would be helpful to provide insights into the growth of the graded films. Grain boundary segregation models,^[^ ^34^ ^]^ together with a change of composition (chemical potential), could be considered as starting points, since a change in thermodynamic parameters directly affects the segregation at GBs. A better understanding of these processes could further optimize the graded film design, leading to fewer AOGs and enhanced material properties.

**Figure 3:**
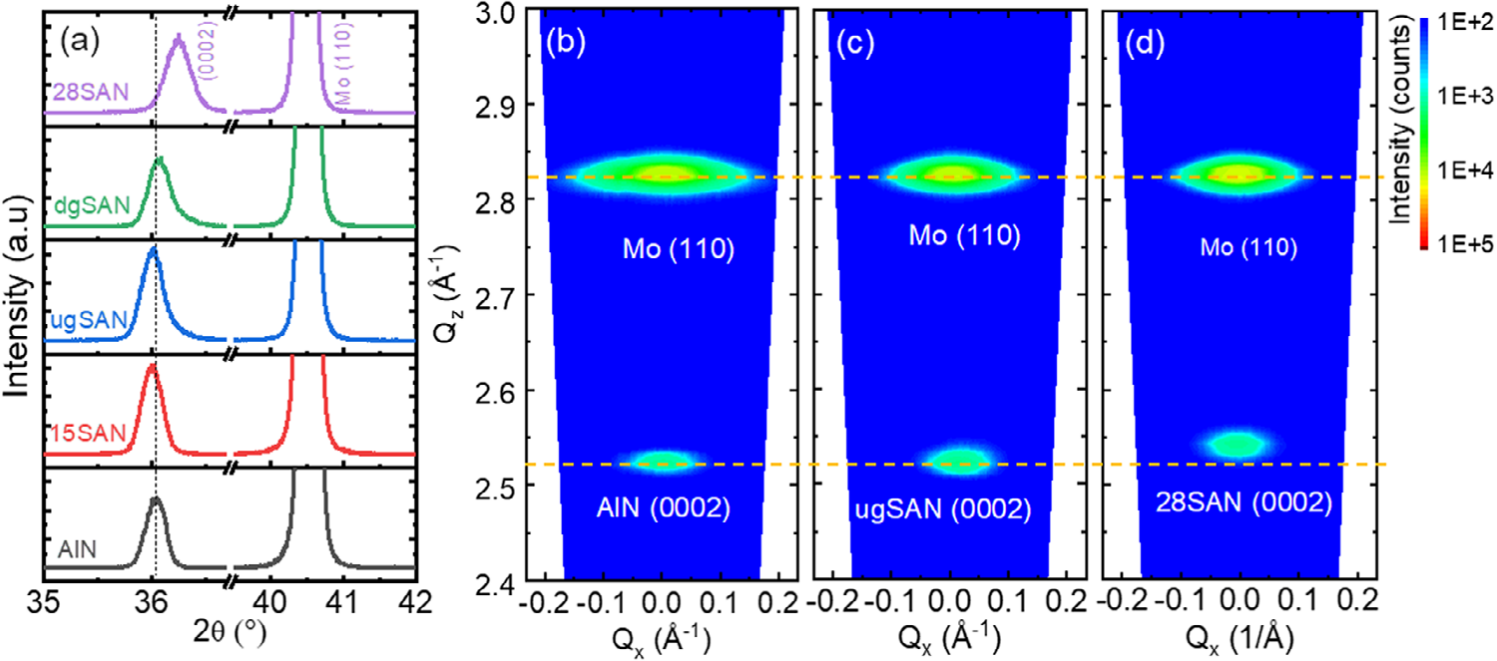
a) *θ*/2*θ* scans for AlN, 15SAN, ugSAN, dgSAN, and 28SAN samples; b–d) Symmetric RSM measurements of AlN, ugSAN, and 28SAN samples, respectively.

**Figure** **3**a shows *θ*/2*θ* X‐ray diffraction (XRD) scans of AlN, 15SAN, ugSAN, dgSAN, and 28SAN thin films. It is clearly seen that only (0002)‐oriented peaks of nitride films and Mo (110) peak are observed, indicating that the nitride films are highly textured. For AlN, 18SAN, ugSAN, and ugSAN, the (0002)‐oriented peak position is located at ≈36°, whereas the (0002) peak position of the 28SAN film shifts to a higher diffraction angle of 36.2°. This shift toward a higher angle with increasing Sc concentration is consistent with literature.^[^ ^19^ ^]^ Interestingly, the shape of the (0002) peak of AlN, 15SAN, and 28SAN is symmetric, while a shoulder on the right‐hand side is detected in ugSAN and dgSAN samples. A similar feature has been observed in epitaxial HoMnO_3_ thin films that was attributed to a strain gradient induced by oxygen vacancies.^[^ ^8^ ^]^

Furthermore, Figure S7a,b (Supporting Information) presents the rocking curves and the calculated out‐of‐plane lattice parameter for the five samples. All the films exhibit good crystalline structures with FWHM of ≈1.9° for AlN, ≈1.6° for 15SAN, ≈1.7° for ugSAN, ≈1.9° for dgSAN, and ≈1.7° for 28SAN samples. The out‐of‐plane lattice parameter *c* of ≈5.24 Å remains unchanged for the AlN, 18SAN, ugSAN, and dgSAN thin films, whereas it reaches ≈5.22 Å in the 28SAN thin film.

To further investigate if a strain gradient plays a role in the graded nitride systems, a symmetric reciprocal space mapping (RSM) was carried out. Figure 3b–d displays RSM pictures of the nitride (0002) and Mo (110) peaks for the AlN, ugSAN, and 28SAN samples. The RSM images for the 15SAN and dgSAN samples are provided in Figure S7c,d (Supporting Information), respectively. All the nitride thin films are single‐phase and fully relaxed. The (0002) intensity peak of the 28SAN sample is located at a higher out‐of‐plane (*Q_z_*) position as compared to other samples, which is consistent with the XRD data shown in Figure 3a. The (0002) peak intensity distributions in reciprocal space are relatively similar among the homogeneous and compositionally graded thin films. Specifically, the width of (0002) peak in the *Q_z_* direction is ≈0.026 for AlN, 0.029 (Å^−1^) for 15SAN samples, 0.029 for ugSAN, 0.031 for dgSAN, 0.031 for 28SAN. This indicates that the out‐of‐plane strain gradient does not play a role in the ugSAN and dgSAN thin films. Therefore, the shoulder seen in the XRD data is attributed to the Sc_0.28_Al_0.72_N component of the graded films, which aligns to the position of (0002) orientation of the 28SAN sample.

**Figure 4:**
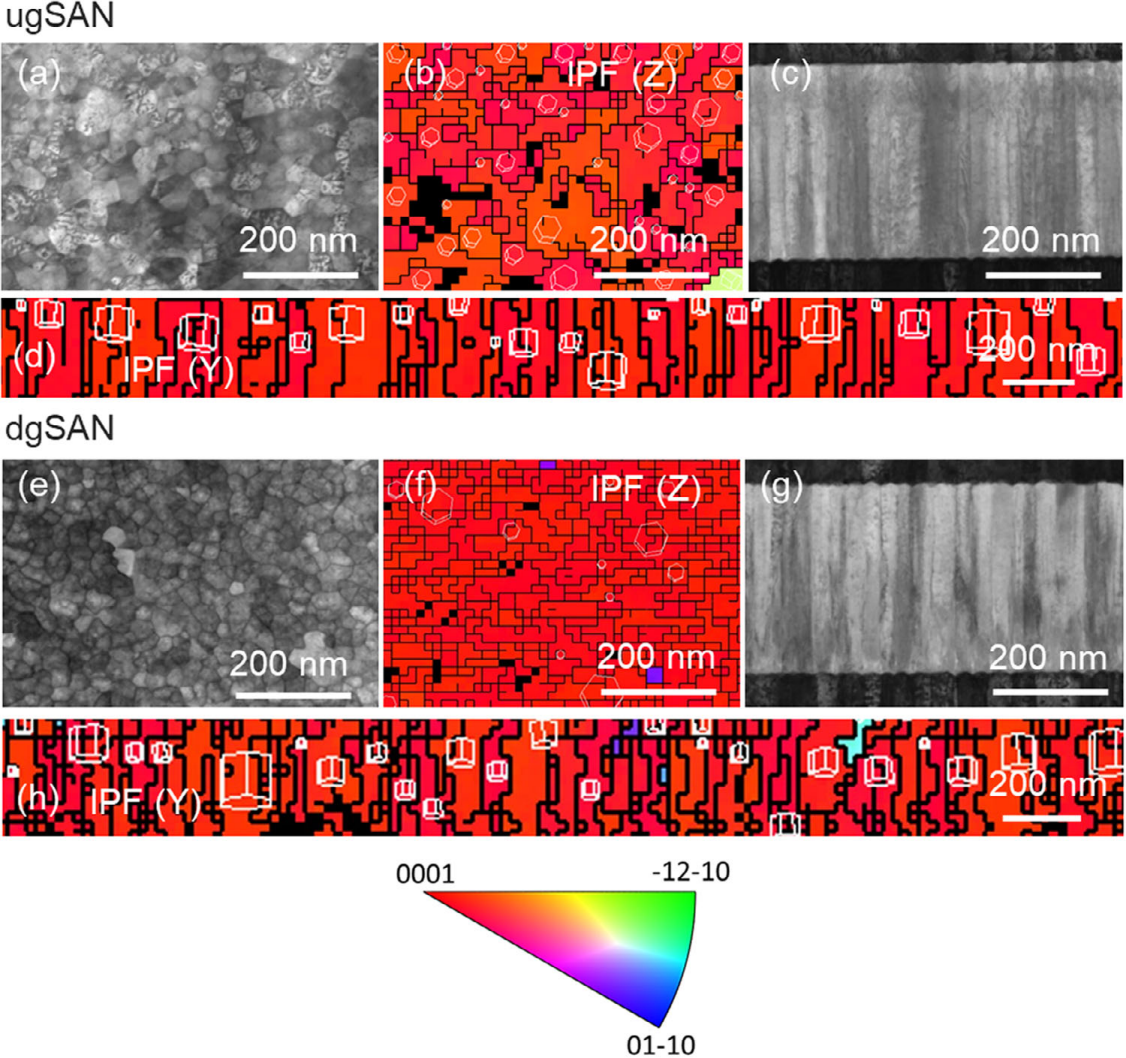
SEM images with TKD maps in plan and cross‐section views analysis of a–d) ugSAN and e–h) dgSAN samples, respectively. Inverse pole figure legend is added. The TKD patterns are indexed with the AlN hexagonal *P63mc* space group. Grains boundaries on TKD images are marked with black lines, schematic orientation of unit cells is shown.

Transmission Kikuchi diffraction (TKD) mapping was carried out to comprehensively study the grain orientations of the graded films. **Figure** **4a–d,e–h** present SEM images and crystallographic mappings on cross‐sectional and plane views for the ugSAN and dgSAN samples, respectively. The results clearly show that the grains in the ugSAN film mainly exhibit the (0001) orientation with a grain‐orientation spread of up to ≈8° in cross‐sectional views (Figure S8, Supporting Information). In contrast, a tiny fraction of in‐plane orientations is observed in the dgSAN film. Utilizing an automated crystallographic mapping technique in TEM, Sandu and co‐workers^[^ ^27^ ^]^ found that the tilted angle between the growth direction and the (0001) crystallographic direction of the AOGs varied between 60 and 90°. It is noted that the face‐to‐face configuration does not apply to co‐sputtering, but the Al and Sc targets are inclined by 30° relative to the horizontal axis. Therefore, the orientation spread of below 8° in the TKD analysis is possibly a result of the incoming flux of adatoms during sputtering.

**Figure 5:**
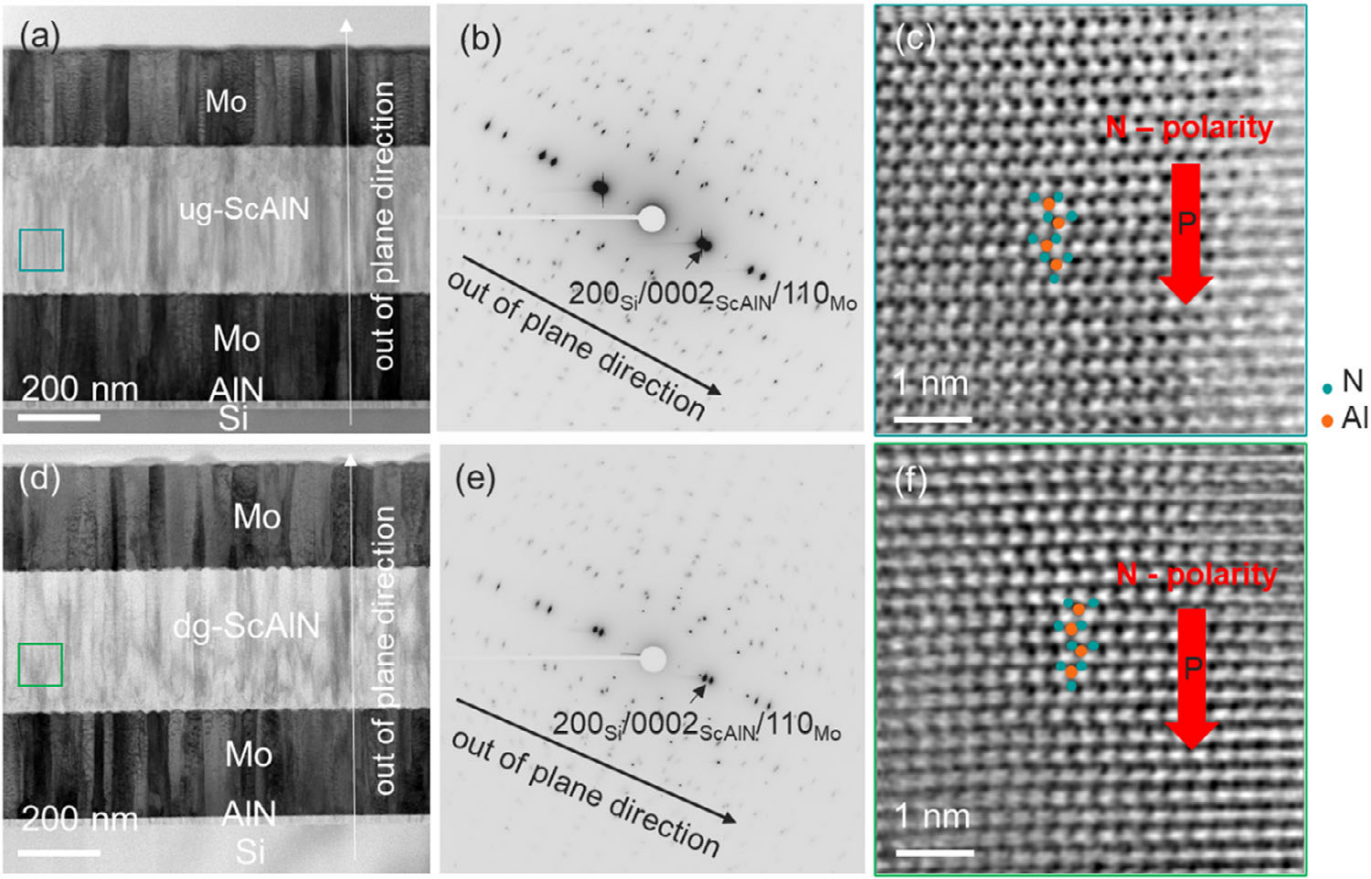
Cross‐sectional BF‐STEM images showing the different layer stack grown on a Si wafer with selected area electron diffraction patterns and high‐resolution STEM micrographs of ScAlN film for a–c) ugSAN and d–f) dgSAN samples. Cyan and green square marks approximate positions where HR‐STEM micrographs were collected for the ugSAN and dgSAN samples, respectively.

TEM and STEM were conducted to investigate the elemental composition, microstructures, and polarity of the graded samples. EDS composition mapping and corresponding EDX lines for the ugSAN and dgSAN samples confirm the elemental composition profile of both graded films, as obtained by ToF‐ERDA shown in Figure S9 (Supporting Information). **Figure** **5**a,c show BF‐STEM micrographs of the ugSAN and dgSAN samples. It can be seen that the microstructures of both graded films display densely packed columnar grains that align to the microstructure of the Mo bottom layer. Two main types of columnar grains, each a few tens of nm in size, are observed and are further confirmed by 4D‐STEM image, as shown in Figure S11a,b (Supporting Information). SAED (selected area electron diffraction) patterns of ugSAN and dgSAN samples are shown in Figure 5b,e, respectively. Well‐defined Sc_x_Al_1‐x_N diffraction spots confirm that the thin films are highly oriented in the [0002] direction for both graded films. Moreover, high‐resolution STEM was executed to study the local polarity of the graded thin films. As shown in Figure 5c,f, both ugSAN and dgSAN thin films exhibit N‐polarity. The direction of polarization across the entire film could not be determined due to the thick sample and the overlap of the grains, which are about a few tens of nm in size.

**Figure 6:**
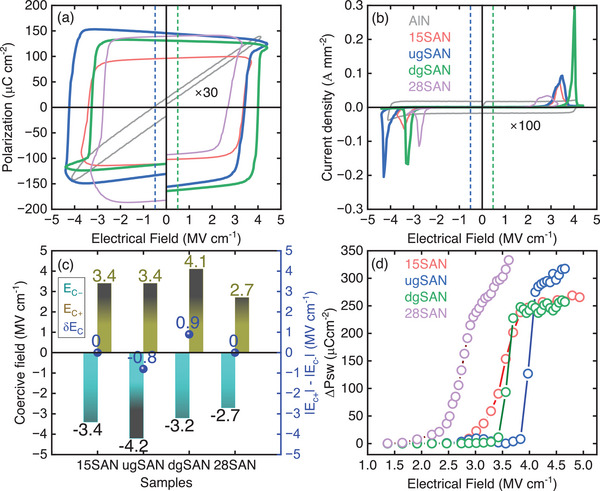
a) Polarization–electric field hysteresis loops measured at 1 kHz and b) corresponding switching current density of the five thin films. For clarity, the *P–E* loop and switching current density of AlN thin film are multiplied by 30 and 100 times, respectively; c) positive and negative coercive fields, and their differences and d) PUND measurements performed for the 15SAN, ugSAN, dgSAN and 28SAN thin films. Blue and green dash lines in a,b) represent a new “0” position, where the *P–E* loop is symmetric, for the ugSAN and dgSAN, respectively.

**Figure** **6**a,b depict polarization–electric field (*P–E*) hysteresis loops and corresponding switching current densities of the five samples, respectively. No ferroelectric switching is obtained for the AlN thin film, whereas both homogeneous and graded thin films exhibit clear ferroelectric behaviors. The *P–E* loops for the homogenous 15SAN and 28SAN films are symmetric, with coercive fields (E_C_) of ≈3.4 and 2.7 MV cm^−1^, respectively. Interestingly, the graded films exhibit highly asymmetric *P–E* characteristics with positive *E_C+_* and negative *E_C‐_* of ≈3.4 and −4.3 MV cm^−1^ for the ugSAN, and ≈4.1 and −3.2 MV cm^−1^ for the dgSAN. Figure 6c summarizes the positive and negative *E_C_* values and the difference between their absolute values. Notably, the *P–E* loops for the ugSAN and dgSAN samples are shifted in opposite directions, with giant built‐in fields (or internal bias/imprint), an average of absolute values of negative and positive electric fields, of 0.4 and 0.45 MV cm^−1^, respectively. It is noted that the increase of polarization versus negative electric field in the ugSAN and dgSAN samples is an artifact due to the charge integration, which is affected by leakage current at high electric fields. This artifact is more profound at lower measurement frequencies and is eliminated at higher measurement frequencies, as shown in Figure S12 (Supporting Information).

A built‐in field has been recently reported in Sc_x_Al_1‐x_N thin films, which can arise from asymmetric electrodes,^[^ ^35^ ^]^ trapped charges at the ferroelectric/non‐ferroelectric interface due to field cycling,^[^ ^36^ ^]^ and/or chargeable defects in the bulk such as nitrogen vacancies (*V_N_*).^[^ ^37^ ^]^ In this study, identical Mo thin films were used as bottom and top electrodes, and the ferroelectric imprint is exclusively observed in the graded films. This implies that the electrode asymmetry is not the primary cause of the imprint in the graded films. Similar asymmetric *P–E* characteristics have been observed in systems like PbZr_1‐x_Ti_x_O_3_ (PZT) with a built‐in field of ≈200 kV cm^−1[^ ^6^, ^7^ ^]^ and Ba_1‐x_Sr_x_TiO_3_ (BST).^[^ ^9^ ^]^ In these systems, strain, giant polarization, and chemical gradients have been identified as contributing factors to the ferroelectric imprint. For Sc_1‐x_Al_x_N system, to the best of our knowledge, flexoelectric coefficients are not yet known. However, DFT analysis combined with microstructural characterizations^[^ ^38^ ^]^ has evidenced the importance of the chemical environment created by the difference in the covalent and ionic bonding of Al and Sc; ferroelectric properties have a much stronger dependence on the local distortion introduced by Sc than the modification of crystallographic lattice parameters. In addition, Landau–Devonshire studies^[^ ^39^, ^40^ ^]^ showed that lattice strain has a limited ability to change polarization switching, suggesting that the elastic fields play a minor role in Sc_1‐x_Al_x_N than in PZT and BST. Therefore, the built‐in field observed in *P–E* measurements is primarily due to the chemical and the concomitant polarization gradient.

The observed built‐in electric field also has a significant impact on the permittivity (or capacitance) – electrical field (*C–V*) of the graded films, as shown in Figure S13 (Supporting Information). A significant shift of *C–V* measurement along the horizontal axis is recognized in both ugSAN and dgSAN samples with opposite directions, in accordance with the shifts in the *P–E* loops. The dielectric constants of the ugSAN and dgSAN thin films are ≈15, similar to that of the 15SAN, where the Sc content is an average of the Sc content in the ugSAN or dgSAN films.

Remnant polarization (*P_r_*) of the nitride films was quantitatively estimated from positive‐up‐negative‐down (PUND) measurements, as shown in Figure 6d. Well‐saturated switchable polarization curves versus applied electric field are obtained in the 15SAN, ugSAN, and dgSAN thin films. Remnant polarization is ≈125 µC cm^−2^ for the 15SAN and dgSAN thin films and is ≈146 µC cm^−2^ for the ugSAN thin film. The polarization curve of the 28SAN sample does not show the saturation behavior. Remnant polarization is roughly estimated to be ≈125 µC cm^−2^ for the 28SAN thin film. The unsaturated behavior of PUND measurement for the 28SAN sample can be attributed to the leakage contribution.

**Figure 7:**
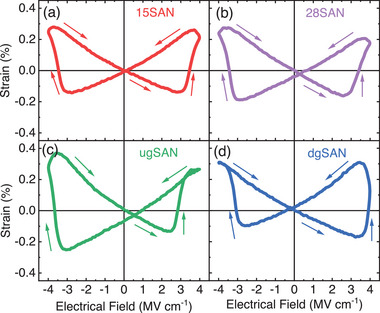
Bipolar electric field–induced strain measurements at 1 kHz of a) 15SAN, b) 28SAN, c) ugSAN, and d) dgSAN thin films.

The graded thin films also exhibit unique electrostrain (*S–E*) behavior, as illustrated in **Figure** **7**. For the homogeneous systems, i.e. 15SAN and 28SAN thin films shown in Figure 7a,b, a symmetric butterfly electrostrain behavior is achieved, with a peak value of ≈0.3% at 3.5 MV cm^−1^. In contrast, the *S–E* curves for the graded films are asymmetric. Figure 7c shows that the electrostrain of the ugSAN thin film significantly reduces in the positive electric field compared to the negative electric field, whereas an opposite trend is observed in the dgSAN thin film following the trend observed in the *P–E* hysteresis measurements.

The asymmetric electrostrain behavior has been reported in several systems, such as (Bi,Na)TiO_3_–BaAlO_2.5_, (BNT–BAO),^[^ ^2^ ^]^ (Bi, Na)TiO_3_–BaTiO_3_–K_0.5_N_0.5_NbO_3_^[^ ^41^ ^]^ and BiFeO_3_–PbTiO_3_–LaFeO_3_ (BFO–PTO–LFO) ceramics.^[^ ^42^ ^]^ In these systems, a similar concept utilizing defect chemistry to form a defect‐engineered morphotropic phase boundary was introduced to generate a giant strain and the asymmetric electrostrain curve. Defect dipoles induced by oxygen vacancies $V_{O}^{..}$ acting as a strong restoring force were attributed to the giant strain due to non–180° domain switching. Meanwhile, the presence of defect dipoles also induces internal bias potentials in ceramics, resulting in asymmetric electrostrain and hysteresis behaviors.^[^ ^2^ ^]^ In the wurtzite ferroelectric Sc_x_Al_1‐x_N system, nitrogen vacancies (*V_N_*) have been identified as a major defect, playing a crucial role in resistive switching^[^ ^37^ ^]^ or polarization switching.^[^ ^43^ ^]^ Yet, defect dipoles related to *V_N_* have never been reported in any III–V wurtzite systems. It is presumed that a point *V_N_* produces a 0D defect–dipole, similar to $V_{O}^{..}$ in BaTiO_3_ system,^[^ ^3^ ^]^ which likely present in all our nitride thin films, as evidenced by ToF–ERDA analysis. Furthermore, if these defect dipoles are the root cause of the asymmetric electrostrain behavior, it would be also appeared in the homogeneous films, i.e. 15SAN and 28SAN. However, the asymmetric behavior is only observed in the graded systems, suggesting that the defect dipoles do not play a role but the chemical/polarization gradient inducing internal bias fields, as confirmed in *P–E* and *C–V* measurements. It could be that defect dipoles in our systems are randomly oriented, thus the total polarization of defect dipoles is zero.^[^ ^44^ ^]^ Therefore, no internal field is induced by defect dipoles, leading to conventionally symmetric *P–E* and *S–E* loops, as observed in the uniform Sc_x_Al_1‐x_N films. Interestingly, a bi‐stable strain state is observed in the graded films, with opposite directions. Specifically, the remanent strain is ≈–0.07 and 0.02% for the ugSAN and dgSAN films, respectively.

The ferroelectric imprint induces two distinct permittivity values (memory window) for both ugSAN and dgSAN films, unlike the homogenous 15SAN and 28SAN films. Recently, Sc_x_Al_1‐x_N thin films have shown promise for high‐temperature non‐volatile memory, essential for modern wide bandgap applications.^[^ ^45^ ^]^ However, their high coercive voltage makes them inefficient for continuous read and write cycles in FeRAM devices. To address this challenge, three‐terminal field effect transistor devices can utilize the permittivity window at zero DC bias, using a small AC signal to read the capacitance state without changing the material's state.^[^ ^46^ ^]^ Here, the graded Sc_x_Al_1‐x_N thin films could hold potential for non‐volatile memory device application, utilizing a new configuration determined by zero‐bias capacitance measurements. In this regard, reducing the thickness of the graded Sc_x_Al_1‐x_N film is required to lower the operation voltage. The existence of bi–stable states could also be of great interest for shape memory actuators and low‐power optical MEMS and RF switches. For instance, electrically‐driven nanolatch mechanisms have been employed to maintain switch‐on states in silicon photonic switches,^[^ ^47^ ^]^ RF‐MEMS switches require voltage for retaining electrical connection through mechanical movement.^[^ ^48^ ^]^

Although a giant built‐in field of 0.4–0.45 MV cm^−1^, which is ≈2.5 times larger than that of PZT or BST graded systems, can be achieved, deterministic control of polarity in the as‐grown films still remains challenging. The plausible explanation is probably due to the higher coercive field of Sc_x_Al_1‐x_N system that makes the graded system more robust to the built‐in field. In PZT or BST systems, the coercive field is below 0.1 MV cm^−1^, allowing a built‐in field of 0.2 MV cm^−1^ to effectively define the initial polarization state. When the Sc concentration increases to 50%, the ferroelectricity of Sc_x_Al_1‐x_N diminishes and the spontaneous polarization decreases from ≈135 to 0 µC cm^2^. Therefore, further increasing the built‐in field in the graded Sc_x_Al_1‐x_N thin films could be achieved due to larger chemical and polarization gradients. As a result, it would become possible to define the initial polarization state. Investigations of composition‐graded Sc_x_Al_1‐x_N thin films with Sc content above 30% are the subject of future research works.

## 3 Conclusion

In summary, we have demonstrated that compositionally graded Sc_x_Al_1‐x_N thin films, grown using a scalable and CMOS‐compatible sputtering process, provide an effective approach to tune both the morphologic and microstructure structures, as well as the dielectric and ferroelectric properties of the films. While homogeneous thin films with 15 and 28% Sc concentrations exhibited a high fraction area (8–10%) of AOGs, the graded thin films significantly suppressed the AOG growth to below 0.2%. This suppression could primarily be attributed to the smooth variation of the in‐plane lattice parameter, which reduces structural defects, then Sc segregation and subsequent AOG nucleation. High‐resolution STEM confirmed that the films have high‐quality columnar structures with locally displayed N‐polarity for both graded thin films. Transmission Kikuchi diffraction further verified that these columnar structures show [0002] orientation with an orientation spread of ≈8. Remarkably, the up‐graded and down‐graded thin films exhibited giant built‐in fields of 0.4 and 0.45 MV cm^−1^, respectively, resulting in opposite horizontal shifts of the hysteresis loops. Microstructural characterizations have evidenced that the polarization/chemical gradient is primarily responsible for the presence of giant built‐in fields in the graded Sc_x_Al_1‐x_N films. This positions the graded nitride system as an archetypical example where the contribution of strain gradient (flexoelectricity) can be excluded. Additionally, the graded Sc_x_Al_1‐x_N thin films also manifested bi‐stable states in the *C–V* and electrostrain characteristics. Our compositionally graded approach, using CMOS‐compatible sputtering technique, offers an avenue to deterministically manipulate Sc_x_Al_1‐x_N film properties, enabling potential applications in non‐volatile memory applications, photonics, RF and MEMS devices, with simplified design and low‐power operation.

## 4 Experimental Section

### Thin Film Deposition

Five distinct thin‐film types, namely AlN, Sc_0.15_Al_0.85_N (15SAN), an up‐graded film with varying Sc concentration from 0 to 28% (ugSAN), a down‐graded film with varying Sc concentration from 28 to 0% (dgSAN), and Sc_0.28_Al_0.72_N (28SAN) samples, were produced by sputtering techniques. The Evatec CLUSTERLINE 200E was used to deposit the nitride films on 8‐inch 250 nm‐Mo/20 nm‐AlN/Si wafers by employing 100 kHz pulsed DC co‐sputtering with a gas mixture of 20 sccm N_2_ and 4 sccm Ar flows at a substrate temperature of 450^ °^C. Al (5N5) and Sc (3N) targets from Umicore with a diameter of 100 mm were utilized for the deposition process. The thickness of nitride layer was fixed at 365 nm. Prior to the depositions, the chamber was evacuated to a base pressure of 5–8×10^−8^ mbar at 450^ °^C, leading to the production of Mo and nitride thin films of excellent quality with a full width at half maximum (FWHM) below 2° and free of oxygen impurities. The ScAlN film composition was modified by varying the sputtering power of the Sc target, while keeping the power of Al target fixed at 800 W. The graded films consist of 18 layers with discrete Sc concentrations, which was varied with an interval of 1–2%. There was no shutter interruption, nor plasma turn‐off when tuning the Sc target power. It is noted that there was no ramping time set for the Sc target power, the ramping time was limited by the power source (ARQ 151). For each type of sample, wafers with and without the top Mo electrode layer were deposited in the same batch with identical process parameters. The entire stack of layers was grown at a chuck temperature of 450^ °^C with no interruption in vacuum.

### Electrical Test Structure Fabrication

First, the dehydration was performed at 180^ °^C for 2 min and followed by HMDS (HexaMethylDiSilazane) adhesion coating. The positive photoresist AZ ECI 3027 (Microchemicals) was spin‐coated at 3000 rpm for 30 s, given a 3 µm thick photoresist. The exposure was carried out on a semi‐automated mask alignment system (EVG@610 Series) using I‐line (365 nm) with a dose of 230 mJ cm^−2^ for 60 s. Then, wafers were developed with AZ 726 MIF developer (Microchemicals), and the pattern was transferred by etching the top Mo layer by Inductively Coupled Plasma – Reactive Ion Etching (ICP‐RIE) (PlasmaPro100, Oxford instruments).^[^ ^49^ ^]^

### Structural and Electrical Characterizations

The film thickness was evaluated via ellipsometry (SE–2000IR, Semilab) in a wide spectral range from 0.5 to 6.5 eV, and was confirmed by cross‐sectional SEM (Helios G4, ThermoFisher). The film morphology was studied by atomic force microscope (AFM, NX20, Park Systems) using the AC160TS 2 nm tip. AFM was carried out on four different positions of 200‐mm wafers at the central, 30, 60, and 90‐mm positions (cf. Supporting Information). The surface roughness and the area fraction of abnormally oriented grains (AOGs) were analyzed on 2 µm × 2 µm AFM micrographs by the Gwyddion software using the threshold function for the detection of AOGs. The symmetric *θ*/2*θ* X‐ray diffraction (XRD) scans, rocking curve measurements, and symmetric reciprocal space mapping (RSM) acquired at the center of wafers were obtained using an X‐ray diffractometer equipped with a 4 bounce Ge 220 monochromator, and a parallel beam X‐ray mirror on the incident path and a PiXcel3D detector (XˊPert MRD XL, PANalytical).

The film composition was determined by Time‐of‐Flight Elastic Recoil Detection Analysis (ToF‐ERDA) at the Tandem laboratory in Uppsala University^[^ ^50^ ^]^ using 36 MeV _127_I^8+^ ion at an incident angle of 67.5° with respect to the sample normal, and the recoils were detected at an angle of 45° in a tandem accelerator. Then the collected ToF‐ERDA data was treated and converted to depth‐profiling atomic concentration using the Potku code.^[^ ^51^ ^]^

Samples for the (scanning) transmission electron microscopy ((S)TEM) were prepared using a Ga+‐source focused ion beam Helios Nanolab 650 HP (Thermo Fischer Scientific, Waltham, MA, USA). For the (S)TEM analysis, cross‐sectional lamellae were prepared, while for the SEM analysis, both cross‐sectional and plan‐view lamellae were prepared.

SEM imaging was performed with a Verios 4G (Thermo Fisher Scientific, Waltham, MA, USA) instrument equipped with a STEM detector generation 3, operated at 30 kV.

Transmission Kikuchi diffraction (TKD) mappings were made in a scanning electron microscope Apreo 2 S (Thermo Fischer Scientific, Waltham, MA, USA) equipped with an electron backscattered diffraction analyzer C‐Swift (Oxford Instruments, Abingdon, UK) at 30 kV. The TKD maps were constructed by indexing diffraction patterns with the space group *P*63*mc* (PDF Card – 00‐025‐1133) in the AZtecCrystal program (Oxford Instruments, Abingdon, UK).

The (S)TEM analysis was performed with a Cs‐corrected ARM200CF (Jeol, Tokyo, Japan) equipped with Energy Dispersive Spectroscopy (EDS) system, operated at 200 kV. The 4D‐STEM dataset was acquired with a Merlin pixelated detector (Quantum Detectors, Oxford, UK).

Electrical characterizations were carried out on Double‐Beam Laser Interferometer (DBLI, aixACCT system GmbH) on 500‐µm diameter capacitors at 1 kHz. Positive‐up Negative‐down (PUND) measurement was measured with a write pulse of 10 µs, a write pulse rise time of 10 µs, and a read time delay of 1 s.

## Conflict of Interest

The authors declare no conflict of interest.

## Author Contributions

T.N. performed conceptualization, investigation, methodology development, data curation, formal analysis, original draft wrote, reviewed and edited the manuscript, as well as project managements. A.G. supported the conceptualization, data curation, and formal analysis, and equally participated in the review and editing process. T.D.D. supported the conceptualization, data curation, and formal analysis, and shared equal responsibility in the review and editing. M.K. and G.D. were equally responsible for data curation, formal analysis, and manuscript review and editing. N.A. supported the investigation and contributed equally to the review and editing. I.N. also supported the investigation and took part equally in the review and editing process. S.N.K. supported data curation and equally participated in the review and editing. J.M. supported the investigation and contributed equally to the manuscript review and editing. A.B.G. contributed equally to the investigation, methodology, data curation, original draft writing, and review and editing of the manuscript. M.M. and M.D. contributed equally to the review and editing of the manuscript.

## Supporting information



Supporting Information

## Data Availability

The data that support the findings of this study are available from the corresponding author upon reasonable request.
