# Supplementary material for: Large Built‐in Fields and Tunable Ferroelectricity in Composition‐Graded ScAlN Thin Films Deposited by Reactive Sputtering
Source: Adv Sci (Weinh). 2025 Apr 25;12(23):2500611. doi: 10.1002/advs.202500611 (PMC12199381; doi:10.1002/advs.202500611)
Supplement: Supplementary file 1 — Supporting Information [file ADVS-12-2500611-s001.pdf]

ADVANCED SCIENCE
Open Access

Supporting Information

for *Adv. Sci.*, DOI 10.1002/advs.202500611

Large Built-in Fields and Tunable Ferroelectricity in Composition-Graded ScAlN Thin Films Deposited by Reactive Sputtering

Tai Nguyen, Anirban Ghosh, Thang Duy Dao, Maja Koblar, Goran Drazic, Nikolai Andrianov, Iurii Nesterenko, Sanjay Nayak, Joaquin Miranda, Andreja Bencan Golob, Mohssen Moridi and Marco Deluca*

Supporting Information

Large Built-in Fields and Tunable Ferroelectricity in Composition-Graded ScAlN Thin Films Deposited by Reactive Sputtering

Tai Nguyen, Anirban Ghosh, Thang Duy Dao, Maja Koblar, Goran Drazic, Nikolai Andrianov, Iurii Nesterenko, Sanjay Nayak, Joaquin Miranda, Andreja Bencan Golob, Mohssen Moridi, and Marco Deluca*

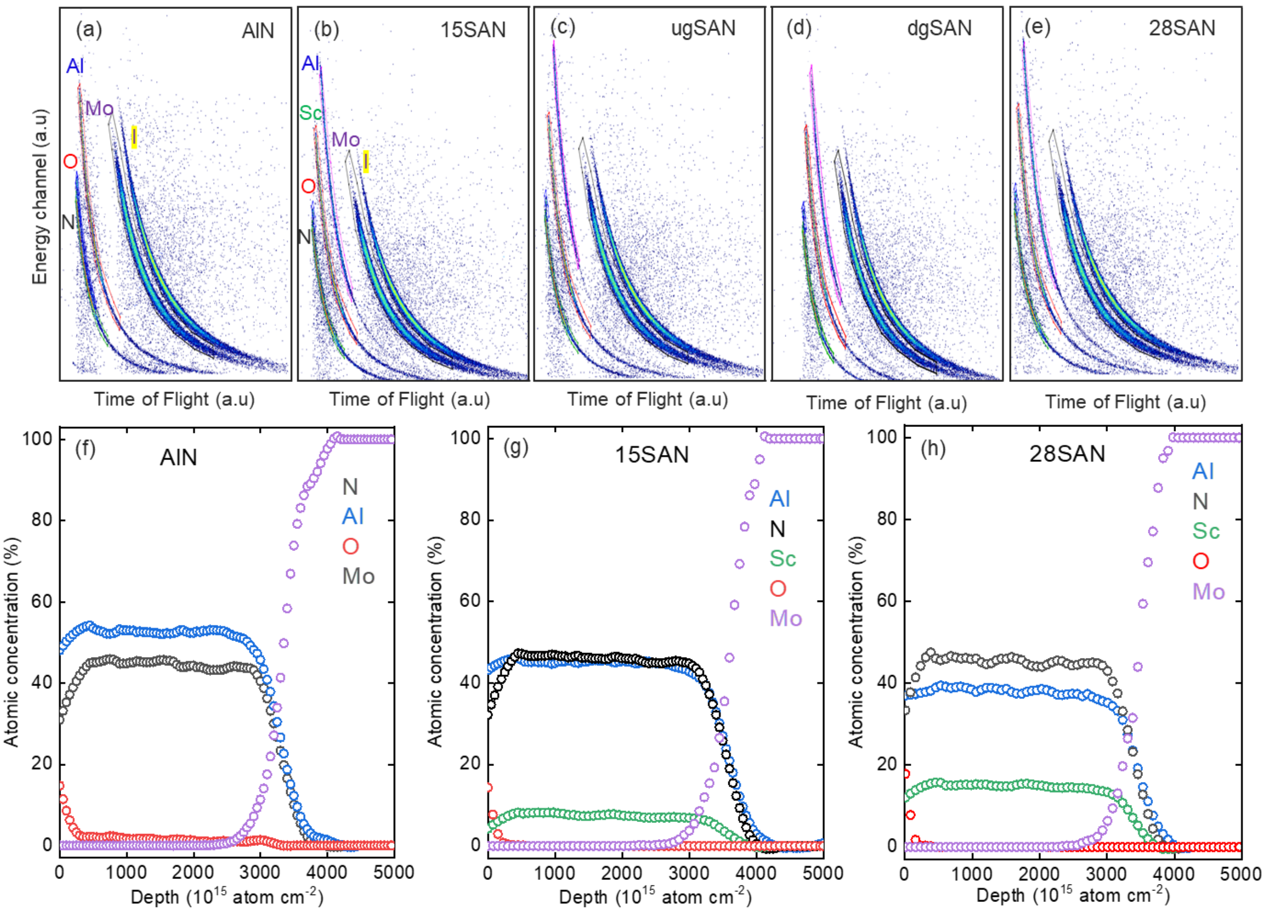

Figure S1: a–e) as-collected ToF-ERDA data of AlN, 15SAN, ugSAN, dgSAN and 28SAN samples, respectively; f–h) relative atomic concentration converted from ToF-ERDA for AlN, 15SAN and 28 SAN samples

Besides the presence of oxygen impurity on the top surface, about 1% of oxygen content in bulk is detected in AlN thin film, as shown in **Figure S1 a and f**. In contrast, oxygen impurity is only observed on the surface for other films, as presented in Figures S1b, h. It is well known that Al and Sc have a strong affinity for oxygen, therefore a native oxide layer is quickly formed when the surface of the nitride layer is exposed to atmospheric environment.

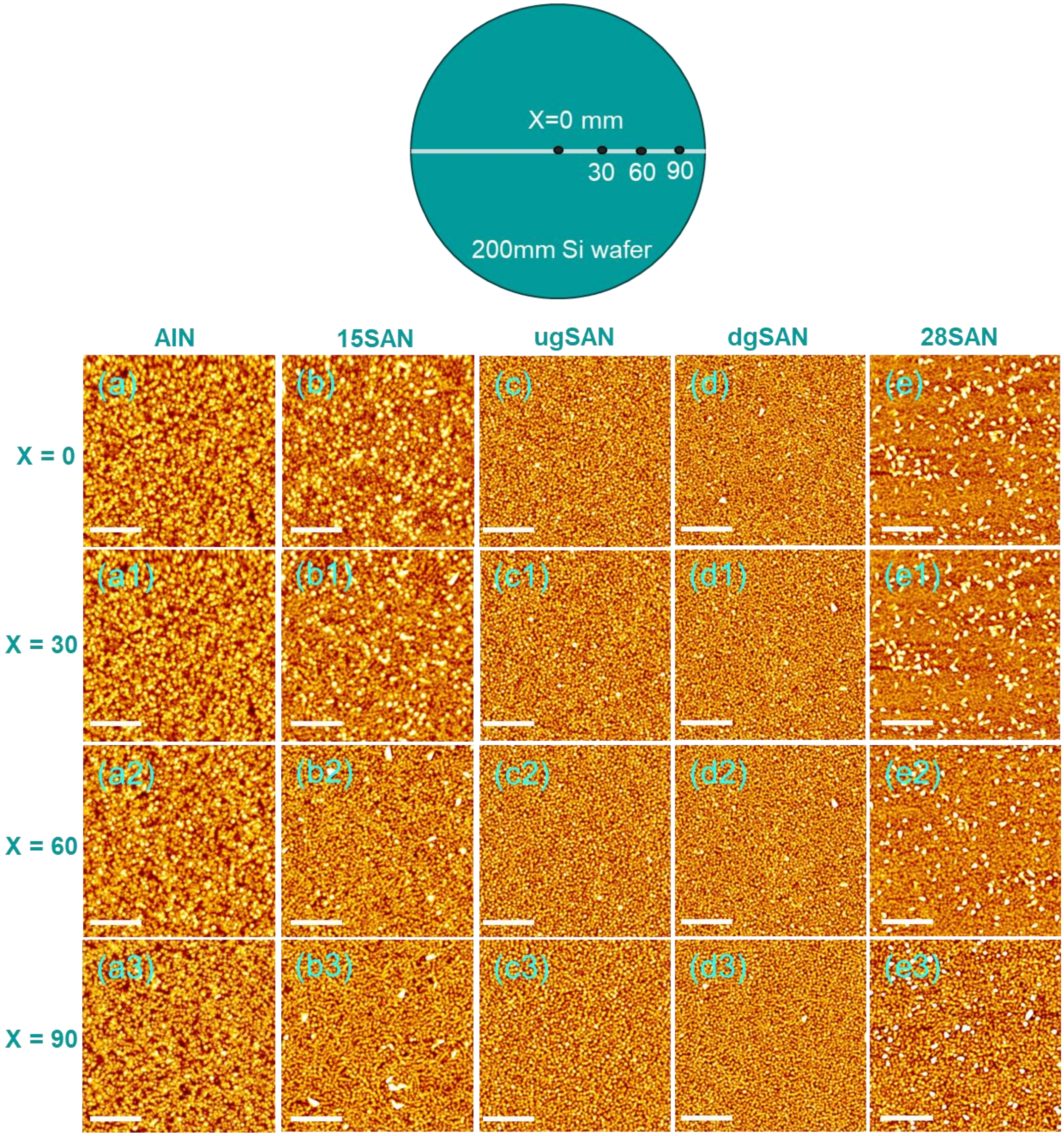

Figure S2: 2μm×2μm AFM micrographs acquired on different position on the 200-mm Si wafer: a-a3) AlN, b-b3) 15SAN, c-c3) ugSAN, d-d3) dgSAN, and e-e3) 28SAN thin films. The scale bar is 500 nm.

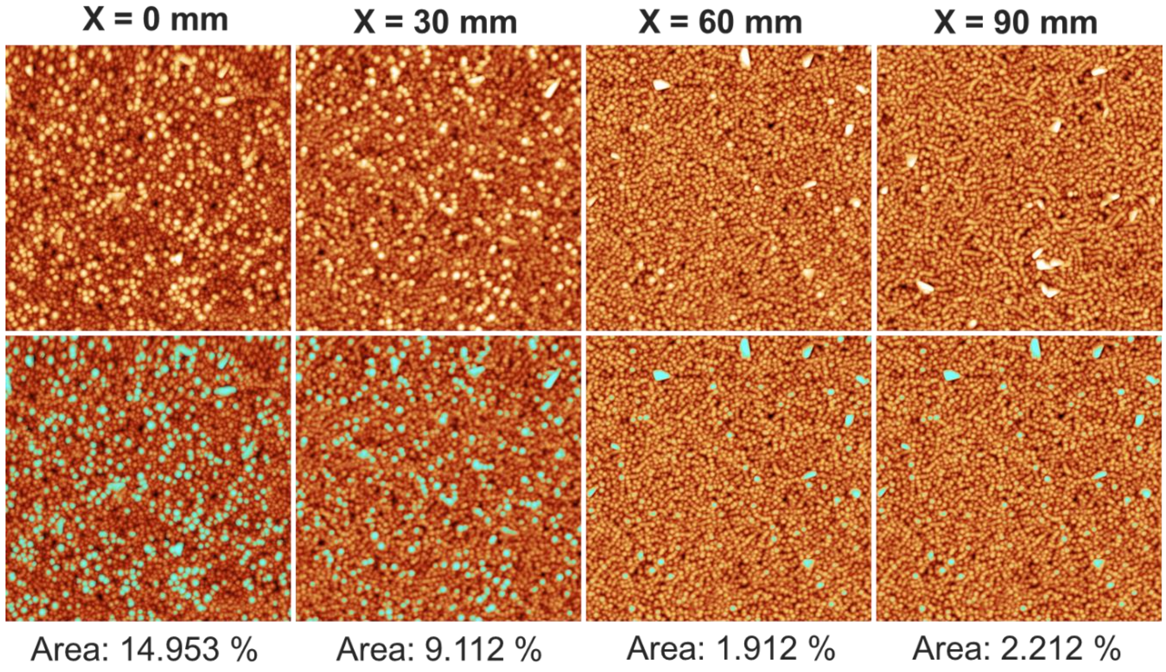

Figure S3: AOGs and corresponding area fraction analysis on 2μm×2μm AFM micrographs for 15SAN thin films. AOGs are marked in cyan.

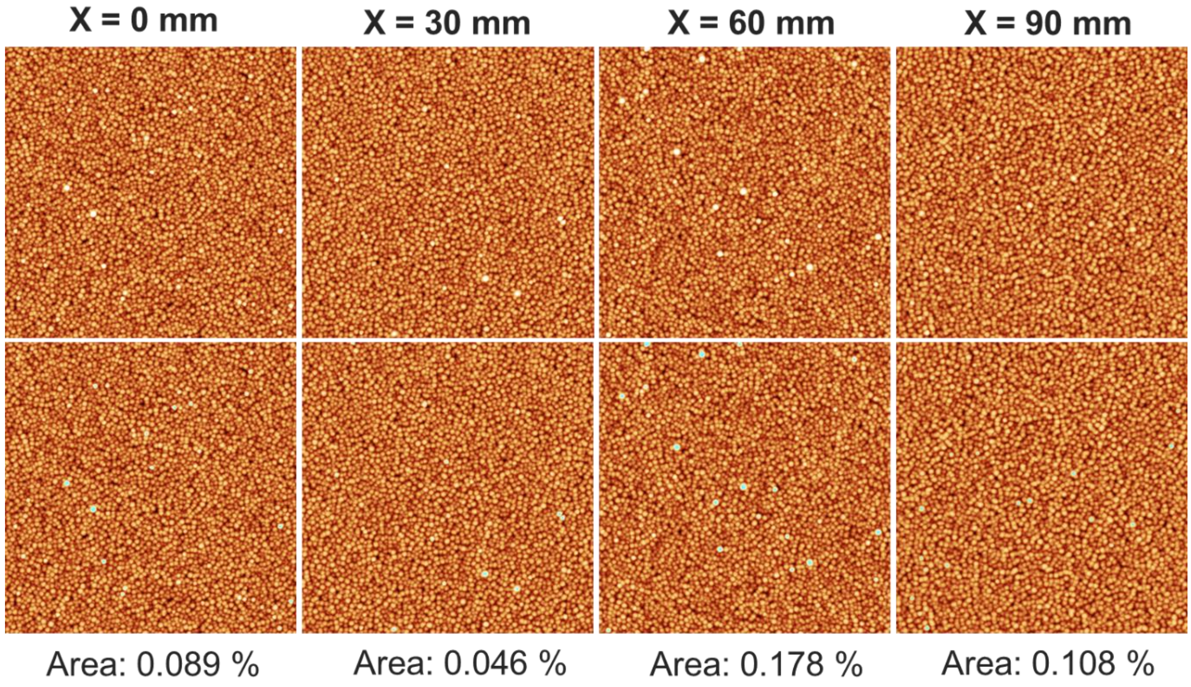

Figure S4: AOGs and corresponding area fraction analysis on 2μm×2μm AFM micrographs for ugSAN thin films. AOGs are marked in cyan.

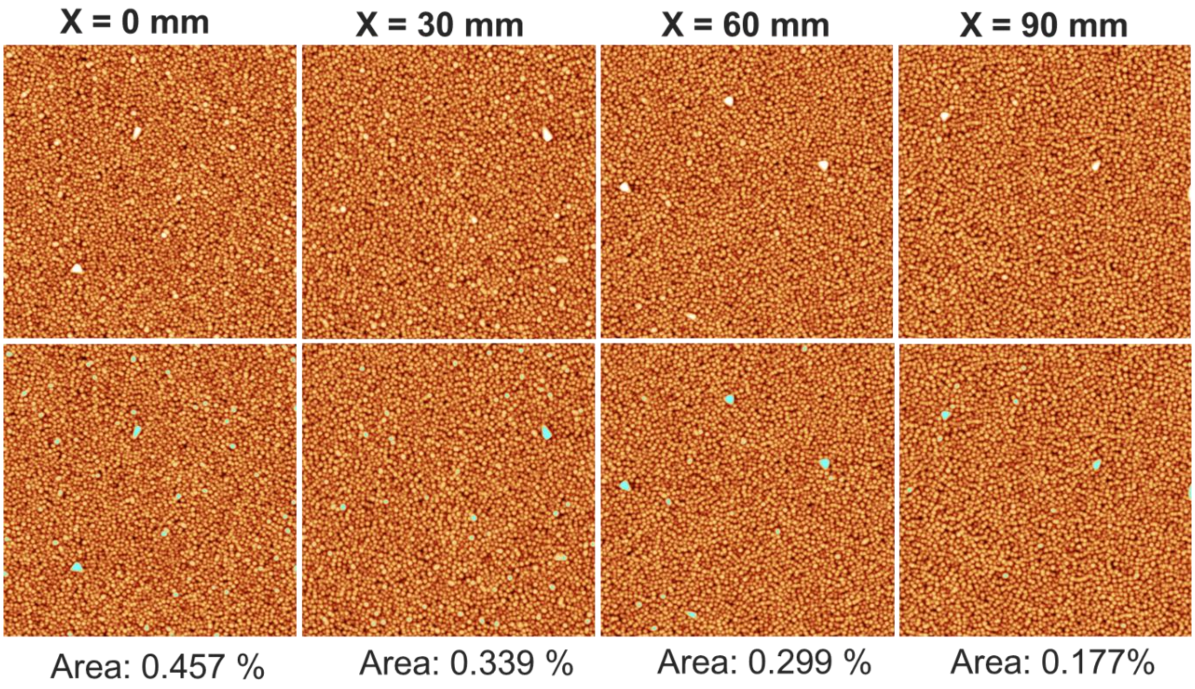

Figure S5: AOGs and corresponding area fraction analysis on 2μm×2μm AFM micrographs for dgSAN thin films. AOGs are marked in cyan.

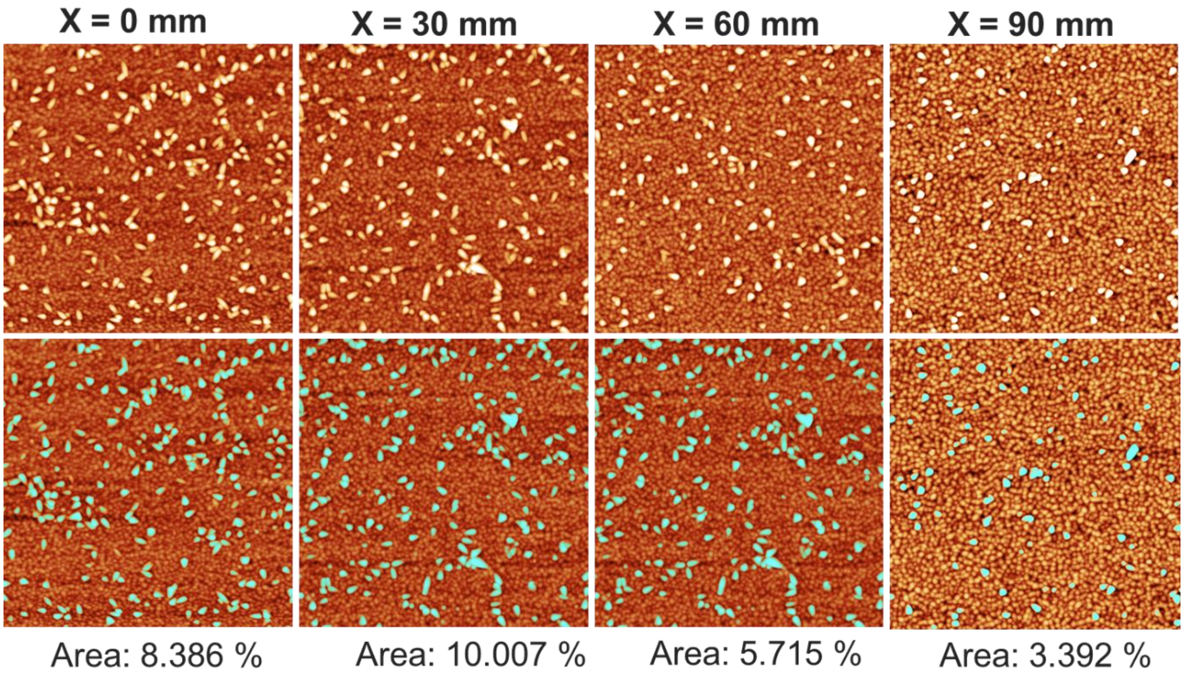

Figure S6: AOGs and corresponding area fraction analysis on 2μm×2μm AFM micrographs for 28SAN thin films. AOGs are marked in cyan.

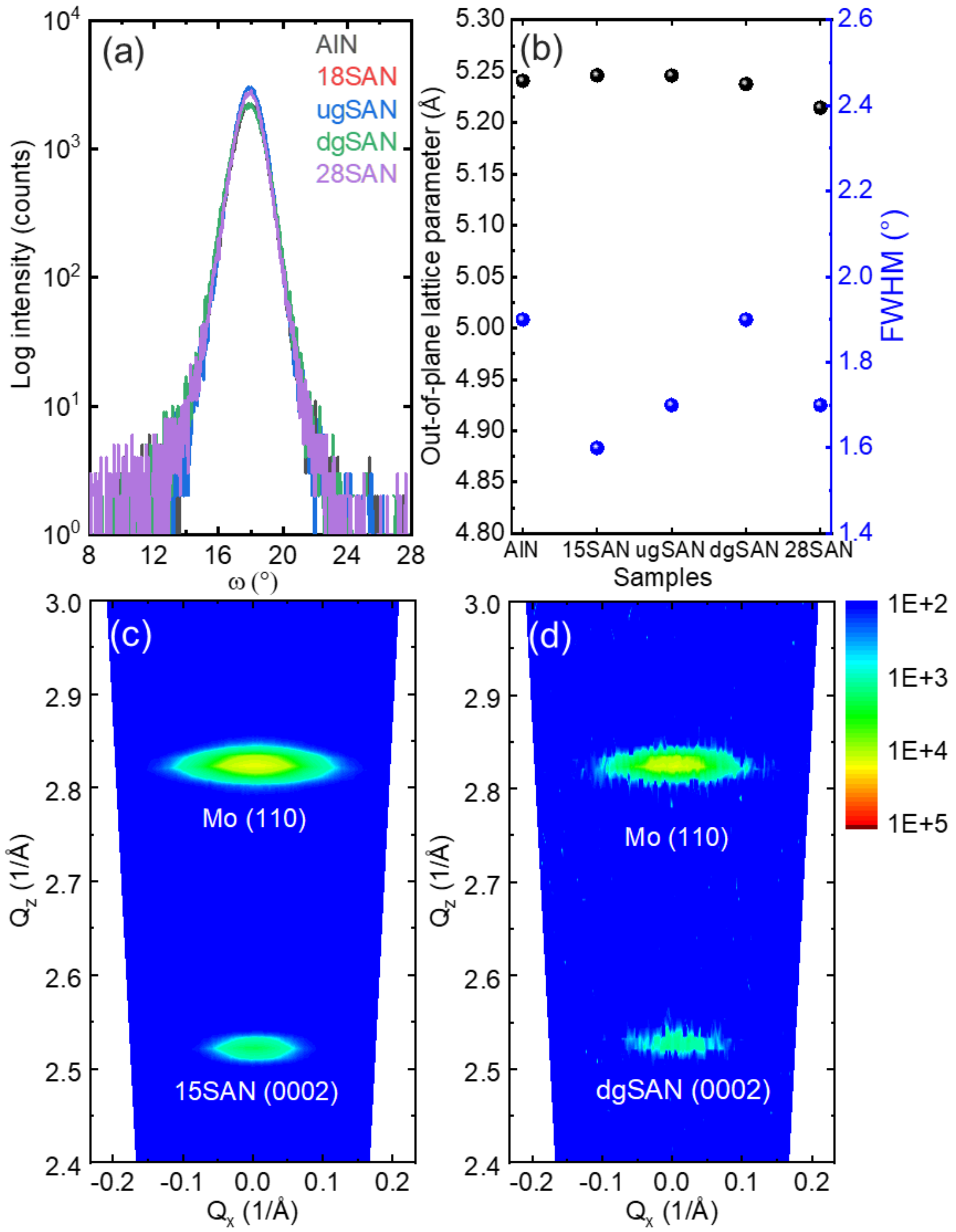

Figure S7: a) Rocking curve measurements and b) calculated out-of-plane lattice parameter and full-width-half-maximum for AlN, 15SAN, ugSAN, dgSAN, and 28SAN samples. c,d) RSM scans of 15SAN and dgSAN films, respectively.

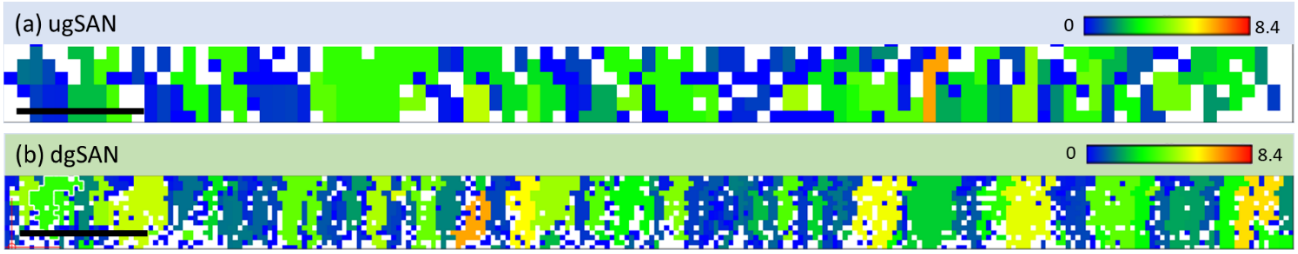

Figure S8: Grain orientation spread from TKD mappings of a) ugSAN and b) dgSAN thin films. The scale bar is 500 nm.

Figure S8 present grain orientation spread (GOS) from transmission Kikuchi diffraction mappings in cross-sectional view for the ugSAN and dgSAN thin films. Both films exhibit (0002) orientation with GOS below 8°.

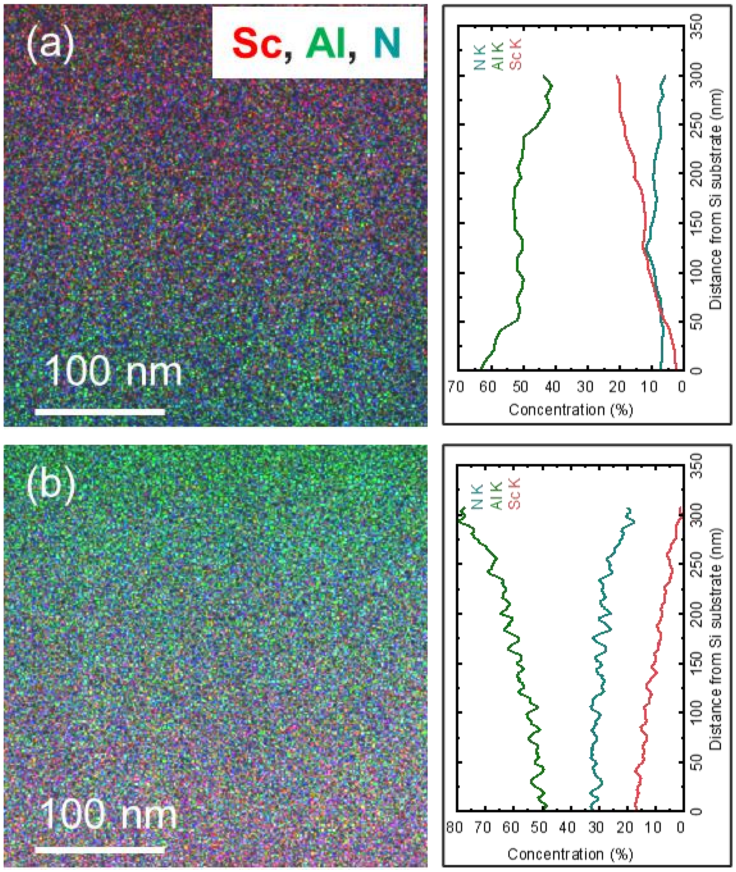

Figure S9: Composition EDS mapping image with corresponding EDX line profile across ScAlN film in a) ugSAN and b) dgSAN. Mapping was obtain using Sc (red), Al (green) and N (blue) K lines.

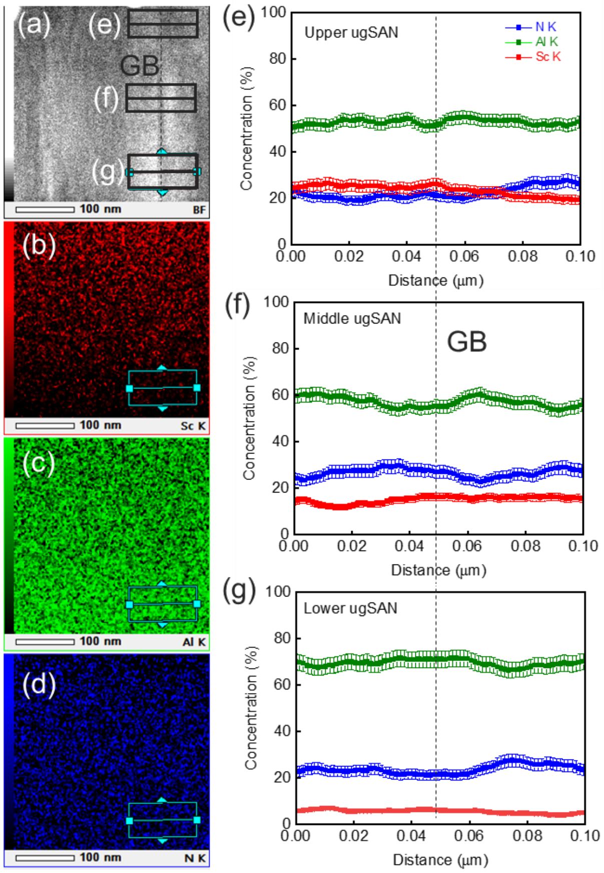

Figure S10: a) STEM image of ugSAN taken between two grains and b-d) corresponding Sc, Al, and N mapping micrographs, respectively. e-g) EDX line profiles of Sc, Al and N across grain boundary (GB) taken at the upper (the high Sc content), middle (the medium Sc content layer) and lower (the low Sc content layer) layers of the ugSAN, respectively. Mapping was obtain using Sc (red), Al (green) and N (blue) K lines. It can be clearly seen that no Sc segregation to GB can be observed within the limits of EDXS analysis.

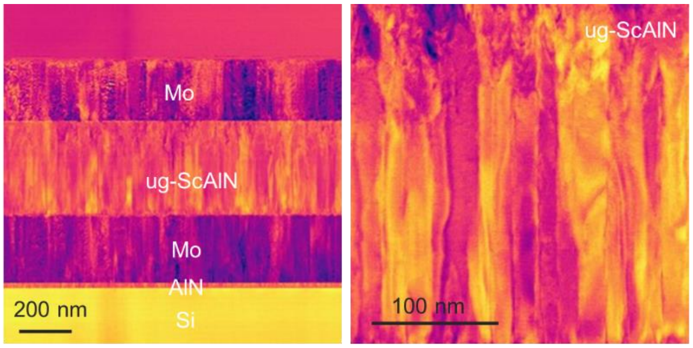

Figure S11: Virtual dark field image from 4D STEM data set of ugSAN sample and corresponding $Sc_xAl_{1-x}N$ film showing columnar grain structure.

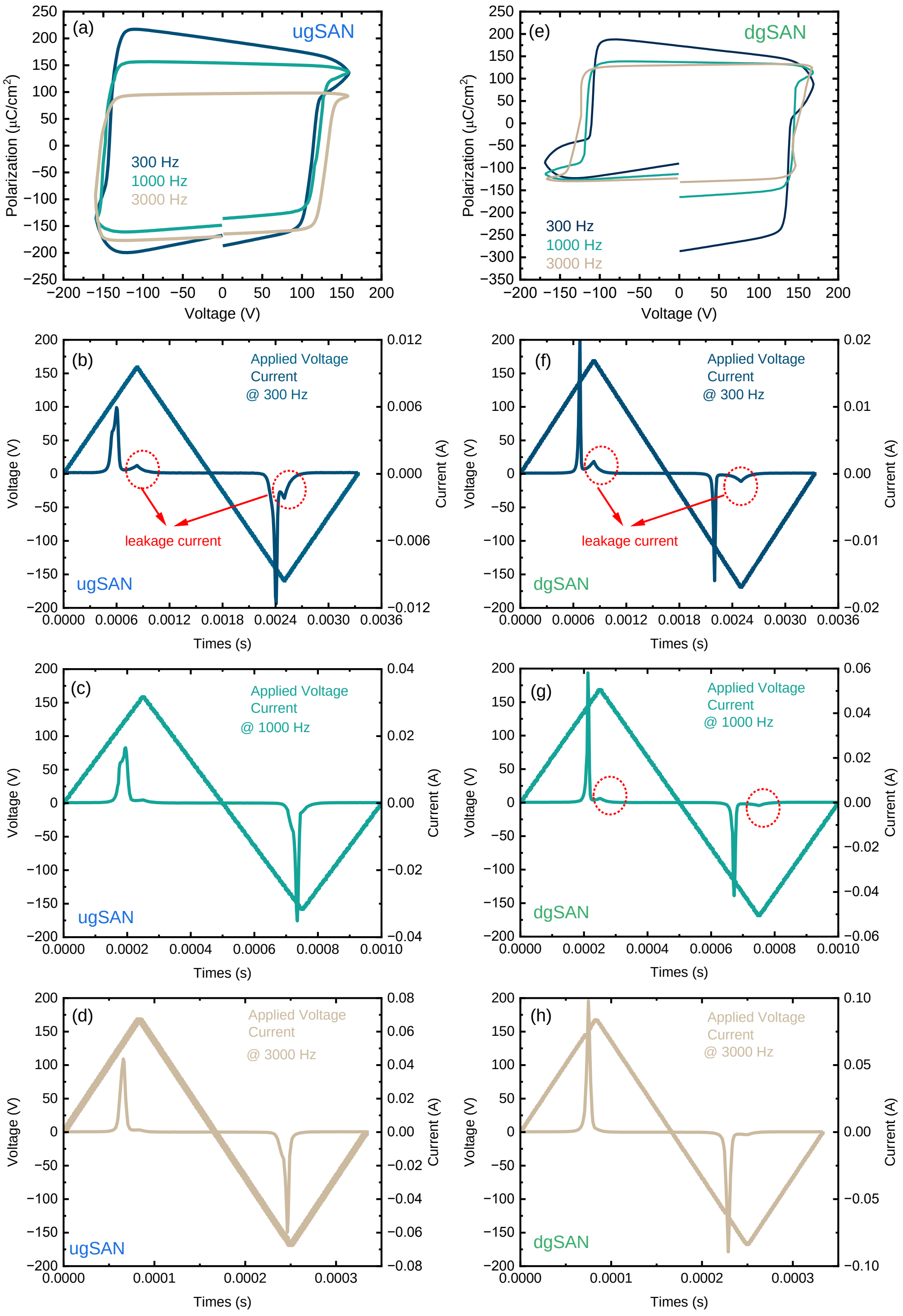

Figure S12: P-E loops measured at different frequencies and IV versus time characteristics: (a-d) the ugSAN and (e-h) dgSAN thin films. It is clearly seen that the negative slopes are decreased at higher measurement frequencies, implying that the contribution of leakage current to the charge integration is eliminated. The elimination of the leakage current can be seen in IV versus time characteristics

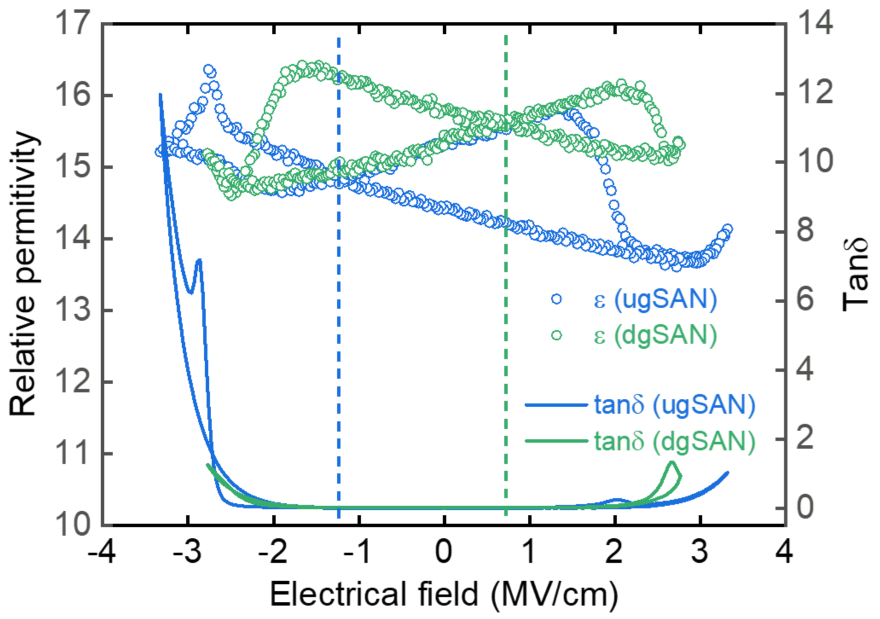

Figure S13: relative permittivity and dielectric loss tan(δ) measurements at 1 kHz showing for ugSAN and dgSAN samples.

Figure S13 displays relative permittivity and dielectric loss tan(δ) measurements for ugSAN and dgSAN samples. Similarly to *P–E* hysteresis and *S–E* bipolar measurements, a built-in field is observed in *C–V* measurements for ugSAN and dgSAN films in the same direction. Namely, built-in fields, an average of negative and positive coercive fields, of ~ -0.6 and ~0.4 MV cm^{-1} are extracted for ugSAN and dgSAN films, respectively.

Calculation of the built-in field induced by polarization gradient

Assuming no presence of free charge, Gauss's law is given: $\varepsilon \frac{\Delta E}{\Delta z} = -\frac{\Delta P}{\Delta z}$; with ε, permittivity, is 15 for the ugSAN and dgSAN; $\frac{\Delta P}{\Delta z}$ and $\frac{\Delta E}{\Delta z}$ are polarization gradient and electrical field gradient induced by polarization gradient along the film thickness, respectively. Thus, the induced electrical field (built-in field) is deduced: $E_{bi} = \frac{\Delta E}{\Delta z} \times t$, with t, thickness of the ugSAN (dgSAN) layer, of 365 nm. Considering theoretical spontaneous polarization of 135 μC/cm^2 for AlN and 125 μC/cm^2 for $Sc_{0.28}Al_{0.72}N$ (28SAN),[1] the calculated E_{bi} is about - 7 MV/cm for the ugSAN and + 7 MV/cm for the dgSAN. The calculated E_{bi} value is overestimated due to the assumption of no presence of free charge carrier that partially subtracts the charges induced by polarization gradient.

Reference

[1] K. Furuta, K. Hirata, S. A. Anggraini, M. Akiyama, M. Uehara, H. Yamada, *J Appl Phys* 2021, *130*, 024104.
